# Assessing Postoperative Quality of Life and Psychological Impact of Free Flap Reconstruction in Advanced Head and Neck Cancer Patients

**DOI:** 10.7759/cureus.71081

**Published:** 2024-10-08

**Authors:** Foteini Neamonitou, Maria Kotrotsiou, Konstantia Papalla, Elpida Mangou, Spyros Stavrianos

**Affiliations:** 1 Plastic Surgery, General Anti-Cancer Oncological Hospital of Athens ‘Agios Savvas’, Athens, GRC; 2 Plastic and Reconstructive Surgery, Evangelismos General Hospital, Athens, GRC; 3 Radiotherapy, General Anti-Cancer Oncological Hospital of Athens ‘Agios Savvas’, Athens, GRC; 4 Oncology, General Anti-Cancer Oncological Hospital of Athens ‘Agios Savvas’, Athens, GRC; 5 Plastic and Reconstructive Surgery, General Anti-Cancer Oncological Hospital of Athens ‘Agios Savvas’, Athens, GRC

**Keywords:** anxiety, depression, free flap reconstruction, head and neck cancer, pain, quality of life, speech, swallow

## Abstract

Introduction

Head and neck cancer (HNC) significantly affects patients worldwide, often resulting in physical disfigurement, functional impairment, and diminished quality of life (QoL). Advanced HNC may necessitate reconstructive surgeries using free tissue transfer, which, despite improving survival, can exacerbate emotional and social challenges such as anxiety, depression, and social isolation. This study aims to evaluate the well-being of patients undergoing free flap surgery for advanced HNC using validated tools.

Methods

We conducted a prospective longitudinal study of 30 patients with advanced HNC (≥T3, stage ΙΙΙ-IV) who underwent free flap reconstructive surgery at the General Oncological Anticancer Hospital of Athens 'Agios Savvas' between January 2018 and January 2024. Patient-reported outcomes were assessed using three validated questionnaires in Greek: the European Organisation for Research and Treatment of Cancer Quality of Life Questionnaire Head and Neck Module (EORTC QLQ-H&N35), the Hospital Anxiety and Depression Scale (HADS), and the Brief Pain Inventory (BPI). Statistical analysis was performed using IBM SPSS Statistics for Windows, version 26.0 (IBM Corp., Armonk, NY), with statistical significance set at p < 0.05.

Results

Among the 30 patients (10 women and 20 men; mean age = 56.6 years), post-surgical assessments indicated significant worsening of symptoms, including increased pain (p < 0.001), swallowing difficulties (p < 0.001), and speech impairments (p < 0.001). Nearly all patients reported increased analgesic use post surgery (p = 0.003), and a significant correlation was found between pain severity and diminished daily functioning, particularly life enjoyment (correlation coefficient = 0.788, p < 0.001). Additionally, 63% of patients (n = 19) were diagnosed with depression and 67% with anxiety (n = 20), with men showing a higher prevalence of depression (p = 0.001). No significant differences in anxiety rates were found between the sexes. Overall, pain severity had a broad impact on both physical and psychological status, contributing to social withdrawal and isolation.

Conclusions

Patients with HNC who undergo free flap reconstructive surgery face significant challenges in emotional, social, and physical well-being. Depression and anxiety are common but often undetected, especially in men. Pain remains a significant factor affecting QoL and social engagement. Comprehensive care models, including standardized pre- and post-treatment assessments, early mental health screenings, and personalized rehabilitation strategies, are essential to improve outcomes and enhance QoL for HNC patients.

## Introduction

Head and neck cancer (HNC) significantly affects patients worldwide, with incidence rates showing an upward trend [1]. Early detection is crucial for achieving optimal outcomes, and a combination of surgical and supportive therapies is often the most effective approach. In advanced stages, palliative or salvage surgery may be required to alleviate pain and discomfort and improve the quality of life (QoL) at the end of life. Unfortunately, these procedures can result in emotional and social difficulties due to disfigurement and functional loss, leading to social isolation and withdrawal [2]. Therefore, comprehensive support, including emotional support, rehabilitation, and counseling, is essential to help patients cope with these challenges.

Patients who undergo oncological and reconstructive surgeries for HNC often experience facial disfigurement and functional impairments. The face is crucial for social perception as it reflects identity, conveys emotions, and produces sounds [3]. Facial disfigurement can impair patients’ ability to articulate speech and express emotions. Additionally, patients may face difficulties with mastication and swallowing, which can necessitate temporary or permanent feeding tubes. These impairments significantly impact QoL and are associated with anxiety, depression, and pain [4]. This study aims to evaluate the well-being of patients who underwent free tissue transfer surgery as part of advanced HNC treatment at a tertiary oncology hospital in Greece.

## Materials and methods

The prospective longitudinal study included all patients diagnosed with advanced HNC (≥T3, stage III-IV) who had undergone curative, palliative, or salvage surgery with free flap reconstruction at the General Oncological Anticancer Hospital of Athens 'Agios Savvas' between January 2018 and January 2024. The study's eligibility criteria required a minimum of one year of follow-up. Unfortunately, out of the 45 patients initially enrolled in the study, 15 did not survive the year and were therefore excluded. We employed three validated Greek-language questionnaires to assess the mental and physical health of the patients. The study was conducted following the Helsinki Declaration and approved by the institutional ethics committee (Institutional Board Approval No.: 7739/F.448/3032021). All participants provided informed consent before their involvement.

Of the 45 patients enrolled, 30 completed all three questionnaires. Surveys were administered during hospital stays, at outpatient clinic follow-ups, or via telephone interviews. The primary survey was the European Organisation for Research and Treatment of Cancer Quality of Life Questionnaire Head and Neck Module (EORTC QLQ-H&N35), a tool designed to assess cancer patients’ physical, psychological, and social well-being. This tool consists of seven scales (pain, swallowing, senses, speech, social eating, social interaction, and sexuality) and 11 individual items addressing issues such as dental problems, restricted mouth opening, dry mouth, sticky saliva, cough, general malaise, use of pain medications, nutritional supplements, feeding tubes, and weight changes [5]. The Greek version of the EORTC QLQ-H&N35 was validated in 2010 [6].

The second survey used was the Hospital Anxiety and Depression Scale (HADS), a 14-item questionnaire designed to measure the severity of anxiety and depression symptoms. It includes two subscales, one for anxiety and one for depression, with items assessing anhedonia, autonomic anxiety, tension, and restlessness [7,8]. The HADS is not a diagnostic tool but helps identify hospital patients who may require further psychiatric evaluation [9]. The Greek version of the HADS was validated in 2008 [10].

The third survey used was the Brief Pain Inventory (BPI), which assesses pain severity and its impact on daily activities, including the location of pain, pain relief, and pain medication usage. The BPI is commonly used in patients with chronic illnesses, such as cancer [11]. The Greek version of the BPI was validated in 2000 [12].

The EORTC QLQ-H&N35 was completed preoperatively and one year postoperatively, while the HADS and BPI were completed one year postoperatively. Statistical analysis was performed using IBM SPSS Statistics for Windows, version 26.0 (IBM Corp., Armonk, NY), and Python 3.11 (Python Software Foundation, Wilmington, Delaware) was used to visualize postoperative changes. Statistical significance was set at p < 0.05.

## Results

We studied 30 patients (10 women and 20 men; mean age = 56.6 years, standard deviation = 11.50 years) who underwent reconstructive surgeries using free flaps following advanced HNC. The types of HNC treated included tongue (27%, n = 8), mandibular (27%, n = 8), retromolar trigone (17%, n = 5), parotid (13%, n = 4), maxillary (13%, n = 4), and lip (3%, n = 1). All patients underwent free flap reconstructive surgery: 44% (n = 13) received a free radial forearm flap, 23% (n = 7) received a free fibula flap, 13% (n = 4) received an anterolateral thigh flap, 10% (n = 3) received a gracilis flap, and 10% (n = 3) received a latissimus dorsi flap (Table 1). All patients received radiation therapy either pre- or postoperatively, and follow-up ranged from 15 months to five years.

**Table 1:** Demographics, cancer location, and reconstruction flap. Demographic information including gender and age, location of HNC, and reconstruction flaps. The data are represented as numbers and percentages. HNC: head and neck cancer.

Patients (N)	30
Men	20
Women	10
Mean age	56.6 years
Head and neck cancer location	
Tongue	8 (27%)
Mandible	8 (27%)
Retromolar trigone	5 (17%)
Parotid gland	4 (13%)
Maxilla	4 (13%)
Lip	1 (3%)
Reconstruction	
Radial forearm free flap	13 (44%)
Free fibular flap	7 (23%)
Anterolateral thigh flap	4 (13%)
Gracilis flap	3 (10%)
Latissimus dorsi flap	3 (10%)

**Figure 1:**
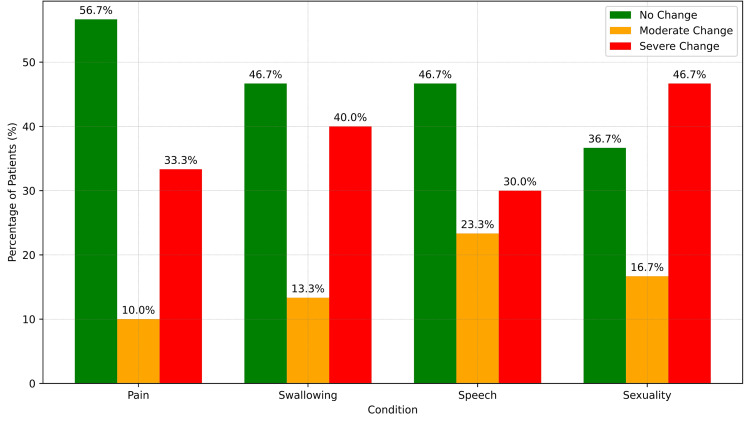
Postoperative changes in pain, speech, swallowing, and sexuality. The data are represented as percentages.

Preoperative EORTC QLQ-H&N35 outcomes showed that 73% of patients (n = 22) reported low pain levels, and 87% (n = 26) had minimal difficulty swallowing. However, postoperative assessments revealed worsening symptoms (Figure 1). All patients reported at least minimal pain, with 30% (n = 9) experiencing severe pain, and many reported challenges with swallowing. Additionally, 47% of patients (n = 14) reported a significant impact on their sexuality and all experienced weight loss. Most patients (93%, n = 28) increased their analgesic use postoperatively to manage discomfort. The Wilcoxon signed-rank test showed statistically significant changes in all symptoms post surgery (Table 2). There was a significant increase in pain, difficulty swallowing, speech impairment, and social isolation related to eating (p < 0.001). Sensory problems also increased (p = 0.004), alongside declines in sexual function (p < 0.001), dental complications, and issues with mouth opening (p < 0.001). Other symptoms included reduced mouth opening (p = 0.001), increased dry mouth (p = 0.003), sticky saliva (p < 0.001), and coughing (p = 0.005). Overall, there was a significant increase in patients’ general feelings of illness (p < 0.001) (Table 3).

**Table 2:** EORTC QLQ-H&N35 scores before and after surgery. This table uses a scale from 1 to 4 to indicate the severity across various domains including pain, swallowing, senses, speech, social eating, social contact, sexuality, problems with teeth, opening mouth, dry mouth, sticky saliva, coughing, and feeling ill. It also includes the number of patients using analgesics and nutritional supplements, having feeding tubes, and experiencing weight changes. The data are represented as numbers. EORTC QLQ-H&N35: European Organisation for Research and Treatment of Cancer Quality of Life Questionnaire Head and Neck Module.

Domains	Severity scale (1-4)	
	Before	After
Pain	1.7	2.5
Swallowing	1.9	2.7
Senses	1.8	2.4
Speech	1.6	2.5
Social eating	1.9	2.6
Social contact	1.9	2.6
Sexuality	1.9	3
Problems with teeth	1.1	2.8
Opening mouth	1.5	2.3
Dry mouth	1.5	2.1
Sticky saliva	1.4	2.3
Coughing	1.2	1.9
Feeling ill	1.6	2.6
	Patients (N = 30)	
Analgesics use	17	28
Nutritional supplements use	0	8
Feeding tube	0	7
Weight gain	0	0
Weight loss	11	30

**Table 3:** Wilcoxon signed rank test. ᵃ Wilcoxon signed rank test. ᵇ Based on negative ranks. The "Based on negative ranks" means that the median post-surgery score was higher than the median pre-surgery score for all variables (consistent with the interpretation that symptoms worsened after surgery). Statistically significant changes were observed in all symptom domains from pre-surgery to post-surgery (p < 0.05).

Analysis	After pain - before pain	After swallowing - before swallowing	After senses - before senses	After speech - before speech	After social eating - before social eating	After social contact - before social contact	After sexuality - before sexuality	After teeth - before teeth	After opening mouth - before opening mouth	After dry mouth - before dry mouth	After sticky saliva - before sticky saliva	After coughing - before coughing	After feeling ill - before feeling ill
Z	-3.307ᵇ	-3.568ᵇ	-2.877ᵇ	-3.573ᵇ	-3.372ᵇ	-3.397ᵇ	-4.021ᵇ	-4.443ᵇ	-3.178ᵇ	-2.966ᵇ	-3.418ᵇ	-2.836ᵇ	-3.338ᵇ
Asymp. Sig. (2-tailed)	0.001	0.000	0.004	0.000	0.001	0.001	0.000	0.000	0.001	0.003	0.001	0.005	0.001

McNemar’s test showed a significant increase in the post-surgical use of analgesics (p = 0.003; Table 4). After surgery, 28 patients needed analgesics compared to only 17 who needed it before surgery (Table 5). According to the HADS, 63% of participants (n = 19) presented with symptoms of depression and 67% (n = 20) with anxiety. Fisher’s exact test was used due to the small sample size, revealing a possible imbalance in depression rates by gender after surgery (p = 0.001; Table 6), with a higher prevalence in men (Table 7). However, anxiety rates did not significantly differ by gender (p > 0.17).

**Table 4:** McNemar's test summary. Asymptotic significances are displayed. The significance level is 0.050. ^a ^The exact significance is displayed for this test (p = 0.003).

Null hypothesis	Test	Sig.	Decision
The distributions of different values across before analgesics and after analgesics are equally likely.	Related-samples McNemar change test	0.003^a^	Reject the null hypothesis.

**Table 5:** Analgesic use before and after surgery. The data are represented as numbers. A total of 11/30 more patients needed analgesics after the surgery.

Analgesics	Before surgery	After surgery
No, n	13	2
Yes, n	17	28

**Table 6:** Fisher's exact test for depression. Fisher’s exact test was used due to the small sample size, revealing a possible imbalance in depression rates by gender after surgery (p = 0.001). df: degrees of freedom; NA: not applicable.

Test	Value	df	Asymptotic significance (2-sided)	Exact Sig. (2-sided)	Exact Sig. (1-sided)
Fisher’s exact test	NA	NA	NA	0.001	0.001
Valid cases, n	30	NA	NA	NA	NA

**Table 7:** Sex depression cross-tabulation. The data are represented as numbers. After surgery, more men (N = 17/20) than women (N = 2/10) suffer from depression.

Sex	Depression		Total, n
	No, n	Yes, n	
Woman	8	2	10
Man	3	17	20
Total	11	19	30

The BPI assessed pain severity (worst, least, average, and current) and pain interference (general activity, mood, walking, work, housework, relationships, sleep, and enjoyment of life). There was a significant sex disparity in “Worst Pain” severity, with men reporting more severe pain (p = 0.024), but no significant sex differences were found in pain impact on daily activities (p > 0.05, Mann-Whitney U Test). Spearman’s correlation coefficient revealed a significant association between pain severity and daily functioning (Table 8), with the strongest correlation observed between pain and life enjoyment (correlation coefficient = 0.7572, p < 0.001). Pain severity was significantly linked with all aspects of functioning, particularly life enjoyment (correlation coefficient = 0.788, p < 0.001). Changes in pain severity significantly affected both physical (p = 0.002) and psychological (p < 0.001) status, indicating widespread impacts on patients’ lives.

**Table 8:** Spearman's correlation between the severity of pain and daily functioning. Significant correlations with all functioning aspects (p < 0.05). Particularly strong link with life enjoyment (r = 0.788, p < 0.001), and an association between pain severity and life satisfaction.

Pain type and severity		Worst pain	Least pain	Pain average	Pain now	Pain severity	General activity	Mood	Walking	Work housework	Relationship people	Relationship pets	Sleep	Enjoyment of life	Pain interference
Worst pain	Correlation coefficient	1.000	0.616	0.655	0.453	0.905	0.520	0.599	0.340	0.629	0.583	0.436	0.604	0.757	0.720
	Sig. (2-tailed)		0.000	0.000	0.012	0.000	0.003	0.000	0.066	0.000	0.001	0.048	0.000	0.000	0.000
Least pain	Correlation coefficient	0.616	1.000	0.557	0.400	0.568	0.507	0.233	0.433	0.462	0.428	0.296	0.470	0.387	0.525
	Sig. (2-tailed)	0.000		0.001	0.028	0.001	0.004	0.215	0.017	0.010	0.018	0.192	0.009	0.035	0.003
Pain average	Correlation coefficient	0.655	0.557	1.000	0.561	0.776	0.476	0.606	0.299	0.628	0.630	0.322	0.720	0.696	0.716
	Sig. (2-tailed)	0.000	0.001		0.001	0.000	0.008	0.002	0.108	0.000	0.000	0.155	0.000	0.000	0.000
Pain now	Correlation coefficient	0.453	0.400	0.561	1.000	0.499	0.721	0.534	0.487	0.516	0.468	0.284	0.523	0.412	0.654
	Sig. (2-tailed)	0.012	0.028	0.001		0.005	0.000	0.002	0.006	0.003	0.009	0.213	0.003	0.024	0.000
Pain severity	Correlation coefficient	0.905	0.568	0.776	0.499	1.000	0.519	0.736	0.353	0.678	0.598	0.362	0.630	0.788	0.762
	Sig. (2-tailed)	0.000	0.001	0.000	0.005		0.003	0.000	0.056	0.000	0.000	0.107	0.000	0.000	0.000
General activity	Correlation coefficient	0.520	0.507	0.476	0.721	0.519	1.000	0.620	0.747	0.618	0.516	0.456	0.446	0.462	0.731
	Sig. (2-tailed)	0.003	0.004	0.008	0.000	0.003		0.000	0.000	0.000	0.004	0.038	0.013	0.010	0.000
Mood	Correlation coefficient	0.599	0.233	0.606	0.534	0.736	0.620	1.000	0.431	0.706	0.548	0.519	0.566	0.760	0.758
	Sig. (2-tailed)	0.000	0.215	0.002	0.002	0.000	0.000		0.017	0.000	0.002	0.016	0.001	0.000	0.000
Walking	Correlation coefficient	0.340	0.433	0.299	0.487	0.353	0.747	0.431	1.000	0.654	0.520	0.632	0.294	0.291	0.610
	Sig. (2-tailed)	0.066	0.017	0.108	0.006	0.056	0.000	0.017		0.000	0.003	0.002	0.114	0.119	0.000
Work housework	Correlation coefficient	0.629	0.462	0.628	0.516	0.678	0.618	0.706	0.654	1.000	0.895	0.769	0.568	0.736	0.931
	Sig. (2-tailed)	0.000	0.010	0.000	0.003	0.000	0.000	0.000	0.000		0.000	0.002	0.001	0.001	0.000
Relationship people	Correlation coefficient	0.583	0.428	0.630	0.468	0.598	0.516	0.548	0.520	0.895	1.000	0.513	0.735	0.736	0.832
	Sig. (2-tailed)	0.001	0.018	0.000	0.009	0.000	0.004	0.002	0.003	0.000		0.010	0.000	0.000	0.000
Relationship pets	Correlation coefficient	0.436	0.296	0.322	0.284	0.362	0.456	0.519	0.632	0.769	0.513	1.000	0.568	0.736	0.705
	Sig. (2-tailed)	0.048	0.192	0.155	0.213	0.107	0.038	0.016	0.002	0.002	0.010		0.001	0.001	0.000
Sleep	Correlation coefficient	0.604	0.470	0.720	0.523	0.630	0.446	0.566	0.294	0.568	0.513	0.568	1.000	0.747	0.737
	Sig. (2-tailed)	0.000	0.009	0.000	0.003	0.000	0.013	0.001	0.114	0.001	0.004	0.001		0.000	0.000
Enjoyment of life	Correlation coefficient	0.757	0.387	0.696	0.412	0.788	0.462	0.760	0.291	0.736	0.735	0.736	0.747	1.000	0.830
	Sig. (2-tailed)	0.000	0.035	0.000	0.024	0.000	0.010	0.000	0.119	0.001	0.000	0.001	0.000		0.000
Pain interference	Correlation coefficient	0.720	0.525	0.716	0.654	0.762	0.731	0.758	0.610	0.931	0.832	0.705	0.737	0.830	1.000
	Sig. (2-tailed)	0.000	0.003	0.000	0.000	0.000	0.000	0.000	0.000	0.000	0.000	0.000	0.000	0.000	

Pain imposes a substantial hindrance on daily activities, with both sexes experiencing elevated anxiety levels and men being more prone to depression symptoms. A significant correlation was identified between pain severity and interference with symptoms of anxiety (p = 0.002) and depression (p = 0.036; Table 9). Notably, the relationship between individuals and their pets remained unaffected.

**Table 9:** Correlations between merged HADS-BPI questionnaires (Spearman’s rho). ** Correlation is significant at the 0.01 level (two-tailed). * Correlation is significant at the 0.05 level (two-tailed). A significant correlation was identified between pain severity and interference with anxiety (p = 0.002) and depression (p = 0.036). HADS: Hospital Anxiety and Depression Scale; BPI: Brief Pain Inventory; NA: not applicable.

Analyte		Depression	Anxiety	Pain severity	Pain interference
Depression	Correlation coefficient	1.000	0.342	0.471**	0.204
	Sig. (2-tailed)	NA	0.064	0.009	0.279
	N	30	30	30	30
Anxiety	Correlation coefficient	0.342	1.000	0.545**	0.384*
	Sig. (2-tailed)	0.064	NA	0.002	0.036
	N	30	30	30	30
Pain severity	Correlation coefficient	0.471**	0.545**	1.000	0.762**
	Sig. (2-tailed)	0.009	0.002	NA	0.000
	N	30	30	30	30
Pain interference	Correlation coefficient	0.204	0.384*	0.762**	1.000
	Sig. (2-tailed)	0.279	0.036	0.000	NA
	N	30	30	30	30

## Discussion

Few studies have examined the QoL of patients undergoing free flap surgery for HNC. Most existing research compares survey results at different time points and correlates them with oncological and survival outcomes [13,14]. Our findings, consistent with other studies, highlight the impact of physical appearance and functional impairments on patients’ daily lives, often resulting in depression, anxiety, social withdrawal, and reduced participation in social activities, such as dining out [15-19]. While we found no significant differences in overall QoL between men and women, except for depression, other studies have reported a general decline in QoL among men compared to women [20,21].

Our study also revealed that pain significantly impacts all aspects of patients’ lives, with an increased use of pain medication following surgery. This underscores the importance of regular and accurate pain assessment to provide effective pain management [22,23]. Notably, our data did not show a correlation between worse outcomes and different age groups, contrasting with a systematic review that reported a decline in QoL with increasing age [24]. Additionally, our study suggests that pet companionship may positively affect well-being in cancer patients, though awareness of these benefits remains low among healthcare providers, and the evidence on risks and benefits is limited [25].

The comparability of studies analyzing QoL in free flap reconstruction for HNC patients is often restricted by significant variability in tumor stages, reconstruction types, survey instruments, and the timing of questionnaires. Furthermore, preoperative assessments are frequently overlooked, complicating comparisons between studies. Prospective studies remain limited [26-28]. We employed a standardized methodology focused on patients with advanced HNC to address these challenges. Our results indicate an urgent need for new strategies and care models to improve QoL and address the specific needs of HNC survivors.

As QoL assessment in HNC patients is still evolving, healthcare professionals must be vigilant in recognizing mental health issues and identifying patients at risk. Early identification of patients needing pain management, speech therapy, occupational therapy, or physiotherapy is essential to ensure timely, individualized treatment, as seen in studies like the INFLUENCE-study (Individualized Follow-Up for Head and Neck Cancer), ultimately enhancing QoL and end-of-life experiences [25,29,30].

Our study has several important limitations. The small sample size of 30 patients limits our findings' statistical power and generalizability. The heterogeneity in tumor staging and types of reconstructive surgeries introduces variability that may affect the interpretation of outcomes. Difficulties in maintaining consistent follow-up challenged the study’s prospective design, as patient attrition occurred due to disease progression, variations in overall survival rates, and logistical barriers in communication. These factors may have introduced selection bias, as patients who were healthier or more engaged in their care were more likely to complete the follow-up assessments. Also, our reliance on self-reported data through validated questionnaires may be prone to recall and reporting biases, as patients may misremember or under-report symptoms, particularly those related to mental health. The lack of preoperative QoL assessments for all patients further limits our ability to fully evaluate the impact of surgical interventions. Additionally, while standardized questionnaires were used, variations in administration methods (in-hospital, outpatient clinic, or telephone interviews) could have influenced patient responses. We also did not control for potential confounders, such as comorbidities, socioeconomic status, and access to rehabilitation services, which could affect outcomes. Lastly, while our study explored the potential positive effects of pet companionship on patient well-being, this was not systematically assessed, and the evidence remains anecdotal. These limitations suggest that our findings should be interpreted with caution, and further research with larger, more diverse cohorts and standardized protocols is necessary to confirm our results and better understand the QoL of HNC patients undergoing free flap reconstruction.

## Conclusions

Patients with HNC frequently experience declines in emotional, social, and physical well-being. Severe cases with symptoms of depression and anxiety often go undetected, particularly among men. Pain is also a significant factor contributing to social withdrawal and isolation. To improve outcomes, standardized pre- and post-treatment care, early screening, and personalized management of mental health disorders are crucial. These approaches can help reintegrate patients into society, reduce stigma, and improve overall QoL.
